# Genome-Wide Association Study Dissects the Genetic Architecture of Pericarp Traits in Fresh-Eating Maize

**DOI:** 10.3390/plants15010074

**Published:** 2025-12-25

**Authors:** Yukun Jin, Song Gao, Huan He, Tong Zhao, Yaohai Yue, Xiangyu Yang, Xinqi Wang

**Affiliations:** Jilin Academy of Agricultural Sciences (Northeast Agricultural Research Center of China), Changchun 130033, China; yukunking@163.com (Y.J.);

**Keywords:** fresh-eating maize, pericarp traits, genome-wide association study, candidate gene

## Abstract

Pericarp characteristics are key factors determining the eating quality of fresh-eating maize. This study aimed to elucidate the genetic basis of traits such as pericarp thickness, break force, and brittleness in fresh-eating maize, identify key genes regulating these traits, and provide a theoretical foundation for improving mouthfeel quality through molecular marker-assisted breeding. Using 196 fresh-eating maize inbred lines with diverse genetic backgrounds, pericarp-related traits were phenotypically measured using a texture analyzer. Genotyping was performed using the GenoBaits Maize 45K Panel chip (MolBreeding, Shijiazhuang City, China). Genome-wide association studies (GWAS) were conducted to identify significantly associated SNP loci, and candidate genes were screened for functional annotation. Phenotypic analysis revealed a significant positive correlation between pericarp thickness and break force, and a significant negative correlation between break force and brittleness. GWAS detected 21, 2, and 1 stable SNPs significantly associated with pericarp thickness, break force, and brittleness, respectively. A total of 47 candidate genes for pericarp thickness, 7 for break force, and 4 for brittleness were identified. Functional annotation indicated that the candidate gene *Zm00001eb314860* (*ZmbZIP130*), annotated as a member of the *bZIP* transcription factor family, may function as a pleiotropic gene involved in regulating pericarp-related traits. These findings demonstrate that pericarp traits in fresh-eating maize are controlled by multiple genes. The significant loci and candidate genes identified in this study lay a foundation for further elucidating the molecular mechanisms underlying pericarp quality formation and for molecular breeding.

## 1. Introduction

Fresh-eating maize, an important global vegetable and grain crop, primarily includes sweet corn (*Zea mays* L. *saccharata* Sturt) and waxy corn (*Zea mays* L. *sinensis* Kulesh) [1]. It is highly favored by consumers due to its rich nutritional value and unique flavor [2]. China has become the world’s largest producer and consumer of fresh-eating maize, with a planting area exceeding 1.73 million hectares, accounting for more than 50% of the global total area [3]. With the upgrading of consumption demands, the market’s requirements for fresh-eating maize quality have shifted from solely pursuing high yield to a comprehensive enhancement of eating quality, nutritional value, and commercial appeal [4].

Eating quality is the core competitiveness of fresh-eating maize, and its superiority is primarily determined by the synergistic effect of pericarp characteristics and endosperm texture. As the outermost layer of the kernel, the pericarp’s thickness, tenderness (measured as break force), and brittleness directly determine the “pericarp residue sensation” during consumption. Studies have shown that varieties with thinner pericarp and higher brittleness are more easily broken during chewing, resulting in a superior texture. In contrast, a thick or tough pericarp leads to a rough chewing experience and reduces consumer acceptance. The endosperm texture is closely related to starch composition [5]. The waxy character of waxy corn is controlled by the *wx* gene located on chromosome 9; its recessive mutation leads to endosperm starch consisting almost entirely of amylopectin [6]. The sweetness of sweet corn originates from endosperm mutant genes such as *su* and *sh2* [7,8], which influence kernel composition by regulating sugar accumulation and starch synthesis pathways [9]. However, compared to internal components like sugars and starch, genetic research on pericarp traits remains relatively weak, particularly lacking a systematic analysis of mechanical properties such as pericarp break force and brittleness [10].

In recent years, genome-wide association studies (GWAS) have become an effective tool for dissecting the genetic basis of complex quantitative traits [11]. This method utilizes linkage disequilibrium (LD) in natural populations, enabling high-throughput gene mapping without constructing segregated populations [12]. In maize research, GWAS has been successfully applied to the genetic dissection of traits like kernel hardness and kernel color, but its application to pericarp traits in fresh-eating maize is still less common [13]. Particularly, research on trait association analysis, screening of key candidate genes, and functional verification remains relatively scarce. Furthermore, traditional GWAS often relies on single-omics data. With the advancement of multi-omics technologies, integrating genomic, transcriptomic, and metabolomic data holds promise for more comprehensively revealing the molecular mechanisms underlying trait formation [14,15].

This study aimed to measure the pericarp traits (thickness, break force, and brittleness) of 196 fresh-eating maize inbred lines using instruments like a texture analyzer, analyze the correlations between different traits, and provide a theoretical basis and germplasm resources for breeding improved eating quality in fresh-eating maize. Current research on maize pericarp tenderness is mostly limited to phenotypic evaluation, with fewer studies at the genetic level. Genetic research on fresh-eating maize started relatively late, particularly creating a gap in the genetic study of pericarp tenderness. The mining and investigation of genes related to the pericarp in fresh-eating maize remain insufficient.

## 2. Results

### 2.1. Descriptive Statistics and Correlation Analysis of Pericarp-Related Traits

**Figure 1:**
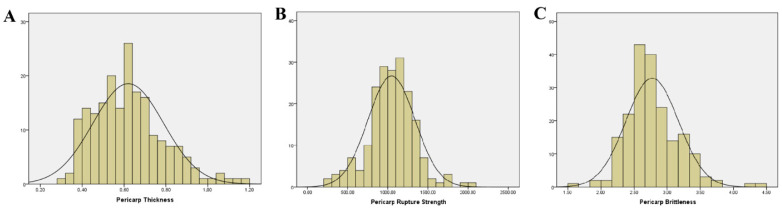
Descriptive statistics of quality traits of different fresh corn. *X*-axis: the variation range of the trait, *y*-axis: the sample count. (**A**): Pericarp Thickness (**B**): Pericarp Rupture Strength (**C**): Pericarp Brittleness.

Based on the mean values of data from 2023 and 2024 for three pericarp-related traits—pericarp thickness, pericarp rupture strength, and pericarp brittleness—a descriptive statistical analysis was performed on 196 fresh-eating maize inbred lines (Table 1, Figure 1). We also calculated the broad-sense heritability for the three traits. Additionally, the mean values of the three traits for the 196 inbred lines in 2023 and 2024 are presented in Supplementary Table S1. The results revealed substantial variation in pericarp thickness and pericarp rupture strength, as indicated by their high coefficients of variation (CV). This variability may significantly impact the sensory quality (e.g., gritty mouthfeel) of fresh-eating maize. Correlation analysis (Table 2) demonstrated a significantly positive correlation between pericarp thickness and pericarp rupture strength, while pericarp rupture strength showed a significantly negative correlation with pericarp brittleness. No significant correlation was observed between pericarp thickness and pericarp brittleness.

### 2.2. Genotyping and Principal Component Analysis of Natural Populations

**Figure 2:**
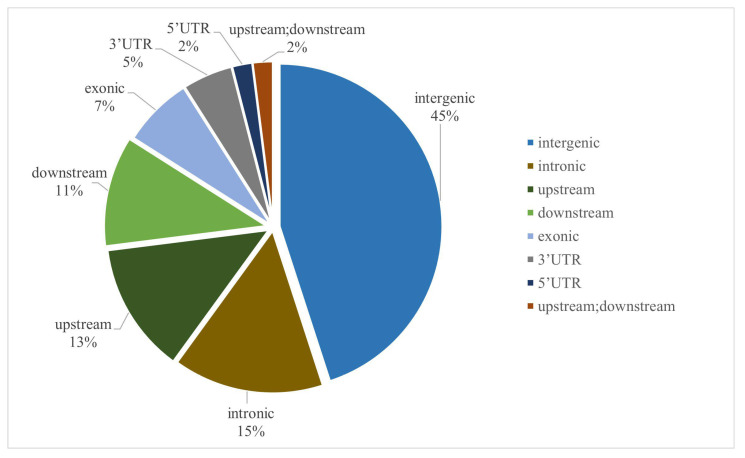
Distribution and functional annotation of genome-wide SNP markers.

Functional annotation of the detected genetic variants was performed based on the genomic locations of variant loci relative to gene positions in the reference genome. As illustrated in Figure 2, intergenic regions accounted for the highest proportion of SNP variations (45% of total SNPs), followed by intronic regions (15% of total SNPs).

**Figure 3:**
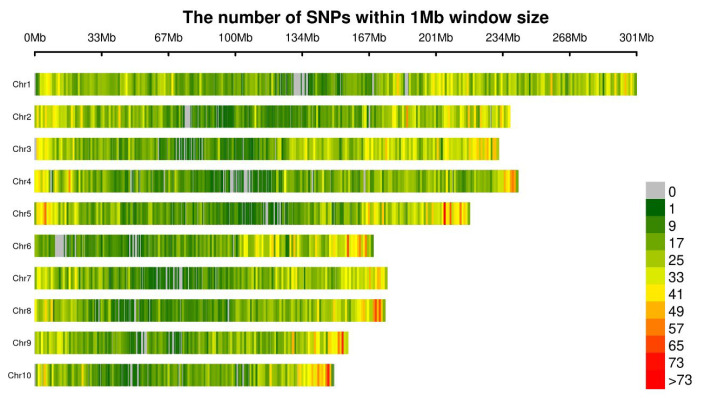
Genome-wide distribution and density of SNP markers. *x*-axis: the physical position (Mb), *y*-axis: the chromosomes (Chr1 to Chr10).

After variant detection and SNP extraction, SNPs with coverage depth < 5× were removed, resulting in a final average of 26,455.3 SNP markers per chromosome used for subsequent analysis. Statistical analysis of SNP distribution and density across each chromosome, based on 264,553 SNP markers processed via R software (v4.3.2), is summarized in Table 3 and visualized in Figure 3. The number of SNPs per chromosome ranged from 19,254 to 40,542. Chromosome 1 contained the highest number of SNPs (40,542 markers), followed by Chromosome 3 (33,280 markers). Chromosomes 6, 8, 9, and 10 exhibited lower SNP counts, with Chromosome 10 having the fewest (19,254 markers). Due to variations in chromosome lengths, SNP density showed minimal variation, averaging approximately 128.43 SNPs per Mbp. Aside from minor localized deviations, SNPs were uniformly distributed across all chromosomes. The highest density (143.32 per Mbp) was observed on Chromosome 3.

**Figure 4:**
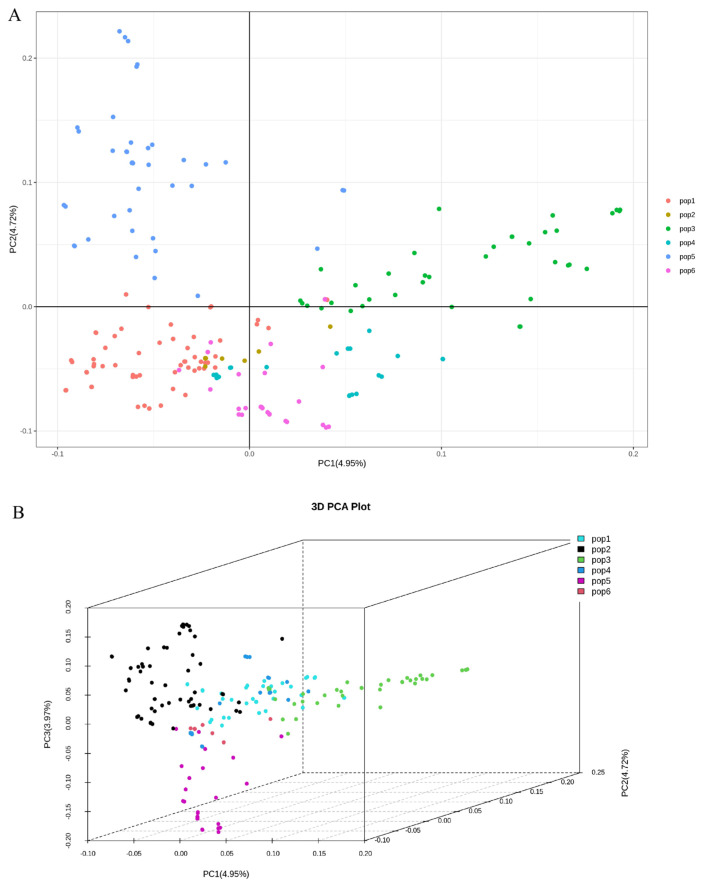
Population structure analysis based on principal component analysis (PCA). (**A**) Two-dimensional and (**B**) three-dimensional PCA plots of 196 fresh-eating maize inbred lines based on 264,553 genome-wide SNPs. PC1, PC2, and PC3 explain 4.95%, 4.72%, and 3.97% of the total genetic variance, respectively.

Principal component analysis (PCA) was conducted on the genotype data of 196 fresh-eating maize inbred lines using TASSEL 5.0 software. Visualization of the top five principal components was performed with R software (v4.3.2). As shown in Figure 4, the dimensionality reduction process clustered the 196 inbred lines into five distinct subpopulations exhibiting distant genetic distances and divergent genetic backgrounds.

### 2.3. Linkage Disequilibrium Analysis in Natural Populations

**Figure 5:**
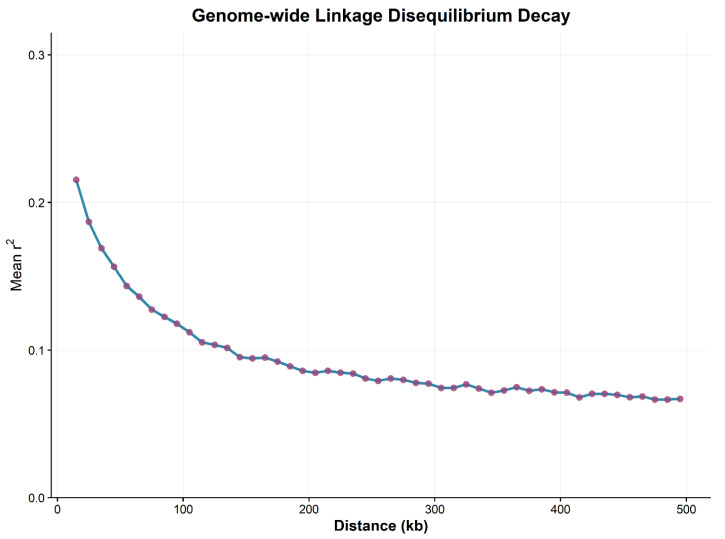
Linkage Disequilibrium Decay Scatter Plot. Scatter plot of pairwise LD against physical distance (kb) between SNPs for all 10 chromosomes.

The marker density required for genome-wide association analysis (GWAS) is determined by the decay distance of linkage disequilibrium (LD), which also governs the precision of association analysis to a certain extent. Using TASSEL 5.0, genome-wide linkage disequilibrium analysis was performed on 264,553 SNP loci screened in the preliminary stage across each chromosome and the entire genome. As shown in Figure 5, in the current population, the vertical coordinate r^2^ represents the recombination and mutation dynamics of LD, which exhibits a declining trend with increasing physical distance. The genome-wide average LD decay distance was determined to be approximately 50 kb when r^2^ decreased to half of its maximum value.

### 2.4. Genome-Wide Association Study and Candidate Gene Identification for Pericarp-Related Traits

Research on pericarp-related traits in fresh-eating maize remains limited at present, particularly regarding gene mining for these traits. Therefore, this study selected three pericarp traits—thickness, rupture strength, and brittleness—for GWAS to identify candidate genes.

#### 2.4.1. Genome-Wide Association Study for Pericarp Thickness

**Figure 6:**
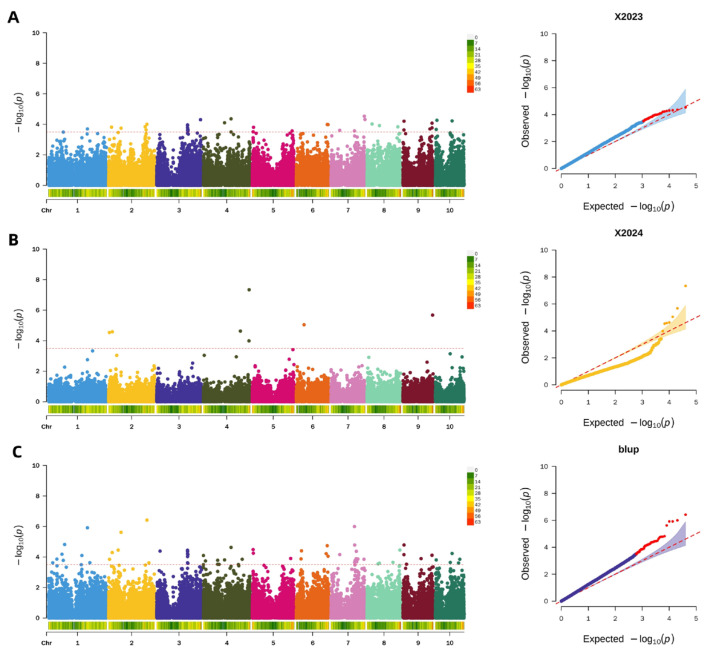
Genome-wide association analysis of maize pericarp thickness. (**A**): Manhattan and QQ plot of maize pericarp thickness in 2023; (**B**): Manhattan and QQ plot of maize pericarp thickness in 2024; (**C**): Manhattan and QQ plot of maize pericarp thickness in BLUP.

A significance threshold of −log_10_(*p*) > 3.5 was applied across all analyses, as it yielded optimal results for detecting significant loci. Using this threshold, GWAS of pericarp thickness identified 36 significant SNPs in the 2023 dataset, which were distributed across all chromosomes. The following year (2024), employing the same criterion, GWAS revealed 7 significant SNPs located on chromosomes 2, 4, 6, and 9. Furthermore, analysis of the BLUP values integrating data from both years identified 67 stable SNPs associated with pericarp thickness, with these loci distributed across all 10 chromosomes. The genomic distributions for each analysis are illustrated in Figure 6.

#### 2.4.2. Screening of Candidate Genes for Pericarp Thickness

Candidate genes were screened within 50 kb upstream and downstream regions of significant SNPs identified by GWAS. For pericarp thickness data in 2023: 96 candidate genes were identified within 50 kb regions flanking 36 significant loci. For pericarp thickness data in 2024: 27 candidate genes were identified near 7 significant loci. For BLUP values of pericarp thickness across two years: 167 candidate genes were identified near 67 significant loci. Overlap analysis: Among the three sets of significant SNPs, 19 overlapping loci were identified. Within their 50 kb flanking regions, 47 candidate genes were ultimately screened (Table 4). Significant SNP and candidate genes detected by GWAS for pericarp thickness.

#### 2.4.3. Genome-Wide Association Study of Pericarp Rupture Strength

**Figure 7:**
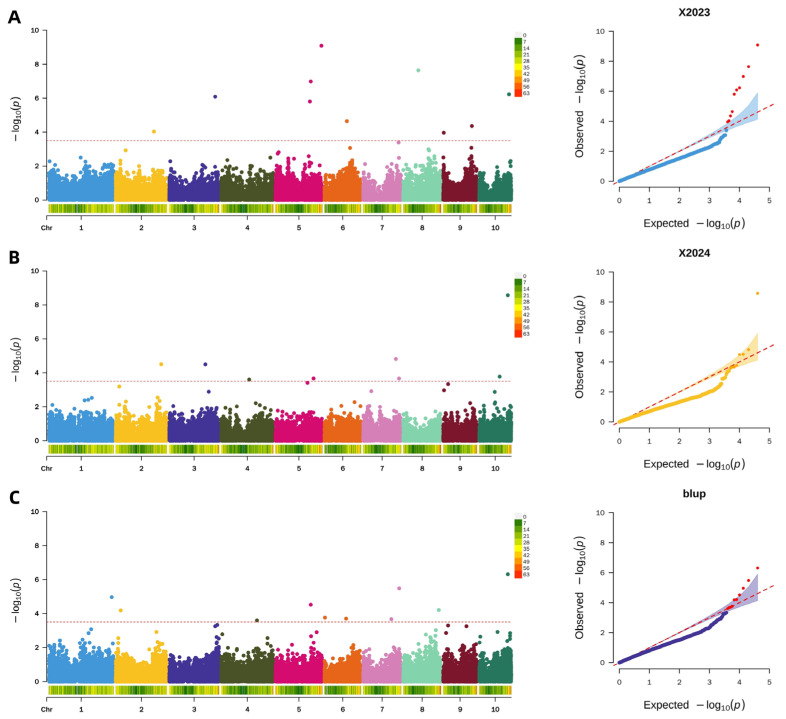
Genome-wide association analysis of maize pericarp rupture strength. (**A**): Manhattan and QQ plot of maize pericarp rupture strength in 2023; (**B**): Manhattan and QQ plot of maize pericarp rupture strength in 2024; (**C**): Manhattan and QQ plot of pericarp rupture strength in BLUP.

A uniform significance threshold of −log_10_(*p*) > 3.5 was applied across all GWAS to identify single nucleotide polymorphisms (SNPs) associated with pericarp rupture strength. In the 2023 dataset, this analysis identified 10 significant SNPs, which were distributed across multiple chromosomes (2, 3, 5, 6, 8, 9, and 10). The subsequent analysis of the 2024 dataset revealed 8 significant SNPs, located on chromosomes 2, 3, 4, 5, 7, and 10. Furthermore, the GWAS performed on the BLUP values, which integrated phenotypic data from both years, detected 10 significant SNPs, indicating stable genetic associations across environments. The results of these analyses are visually summarized in the accompanying Manhattan and QQ plots (Figure 7).

#### 2.4.4. Screening of Candidate Genes for Pericarp Rupture Strength

Candidate genes were screened within 50 kb upstream and downstream regions of significant loci identified by GWAS. For pericarp rupture strength data in 2023: 28 candidate genes were identified within 50 kb regions flanking 10 significant loci. For pericarp rupture strength data in 2024: 33 candidate genes were identified near 8 significant loci. For BLUP values of pericarp rupture strength across two years: 27 candidate genes were identified near 10 significant loci. Overlap analysis: Among the three sets of significant SNPs, 2 overlapping loci (Chr5_163983546 and Chr10_133474083) were identified. Within their 50 kb flanking regions, 7 overlapping candidate genes were ultimately screened (Table 5).

#### 2.4.5. Genome-Wide Association Study of Pericarp Brittleness

**Figure 8:**
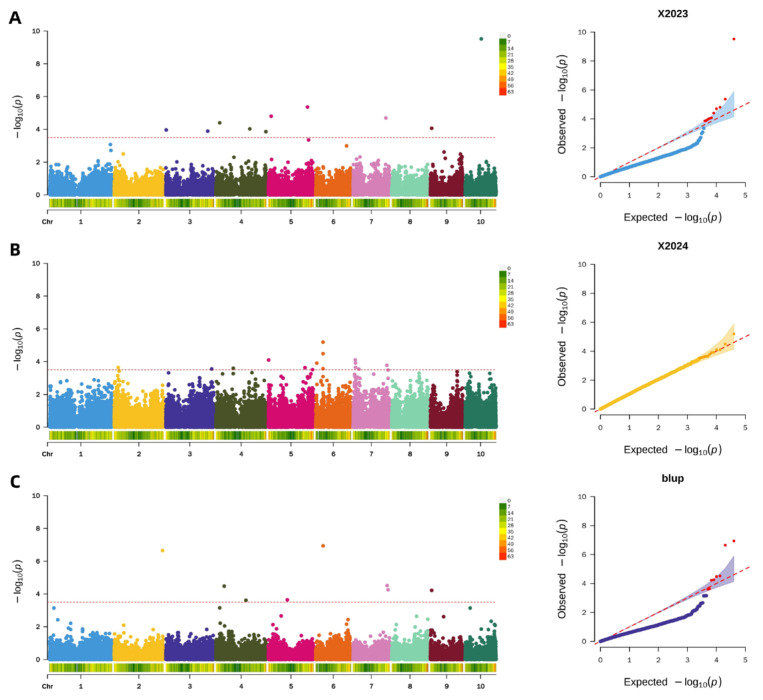
Genome-wide association analysis of maize pericarp brittleness. (**A**): Manhattan and QQ plot of maize pericarp brittleness in 2023; (**B**): Manhattan and QQ plot of maize pericarp brittleness in 2024; (**C**): Manhattan and QQ plot of pericarp brittleness in BLUP.

Applying a uniform significance threshold of −log_10_(*p*) > 3.5, GWAS were conducted to identify genetic loci associated with pericarp brittleness. Analysis of the 2023 dataset revealed 10 significant SNP loci, primarily distributed on chromosomes 3, 4, 5, 7, 9, and 10. The following year (2024), GWAS identified 15 significant loci located on chromosomes 2, 3, 4, 5, 6, and 7. Furthermore, an integrated analysis using BLUP values combining data from both years detected 8 stable SNP loci associated with pericarp brittleness, which were mapped to chromosomes 2, 4, 5, 6, 7, and 9. The results of these analyses are visualized in the corresponding Manhattan and QQ plots (Figure 8).

#### 2.4.6. Screening of Candidate Genes for Pericarp Brittleness

Candidate genes were screened within 50 kb upstream and downstream regions of significant loci identified by GWAS. For pericarp brittleness data in 2023: 20 candidate genes were identified within 50 kb regions flanking 10 significant loci. For pericarp brittleness data in 2024: 34 candidate genes were identified near 15 significant loci. For BLUP values of pericarp brittleness across two years: 27 candidate genes were identified near 8 significant loci. Overlap analysis: Among the three sets of significant SNPs, 1 overlapping locus (Chr9_6307872) was identified. Within its 50 kb flanking region, 4 overlapping candidate genes were ultimately screened (Table 6).

#### 2.4.7. Overlapping Loci for Candidate Genes Associated with Pericarp-Related Traits

Genome-wide association analysis of three pericarp-related traits—pericarp thickness, pericarp rupture strength, and pericarp brittleness—was conducted using both single-year phenotyping data and multi-year Best Linear Unbiased Prediction (BLUP) values. Candidate genes identified from single-year datasets were compared with those derived from BLUP values. This analysis revealed five pleiotropic candidate genes potentially regulating multiple traits: *Zm00001eb314860, Zm00001eb314870, Zm00001eb314880, Zm00001eb415550,* and *Zm00001eb415560* (Table 7).

*Zm00001eb314860* (annotated as *ZmbZIP130*): Member of the *bZIP* transcription factor family. Studies in apples (*Malus domestica*) and tomatoes (*Solanum lycopersicum*) indicate their involvement in starch and sucrose metabolism and glycolytic pathways. Overexpression of *bZIP* in tomato and apple calli significantly increased starch and sucrose content while reducing glucose levels compared to wild-type controls [16].

*Zm00001eb314870*: Identified as a non-coding RNA (ncRNA) via MaizeGDB. Its homologous sequence has been detected in rice (*Oryza sativa*), but functional validation remains unreported.

*Zm00001eb314880*: No known functional annotation currently available.

*Zm00001eb415550* & *Zm00001eb415560* (annotated as *ZmCCR4*): Regulate the synthesis of serine/threonine protein kinase *CCR4* and may participate in biotic/abiotic stress responses in maize.

For the Gene Ontology (GO) functional annotation, we utilized Entrez ID (NCBI Entrez Gene ID) instead of the original gene identifiers due to its uniqueness, stability, and cross-species compatibility, which ensures the accuracy of the analytical results. During the ID conversion process, a portion of the original IDs were filtered out as they failed to find corresponding Entrez IDs. Subsequently, we performed GO functional annotation, analysis specifically on the candidate gene *Zm00001eb314860*, which is potentially involved in both pericarp rupture strength and pericarp thickness. GO functional annotation, of *Zm00001eb314860* indicated its involvement in: Positive regulation of DNA-templated transcription (GO:0045893), Positive regulation of RNA biosynthetic process (GO:2001141), Positive regulation of biosynthetic process (GO:0009891), Positive regulation of macromolecule biosynthetic process (GO:0010558), Positive regulation of cellular biosynthetic process (GO:0031326).

#### 2.4.8. Functional Prioritization and Mechanistic Hypotheses for Candidate Genes

While the initial screening of candidate genes within 50 kb of significant SNPs is a standard practice in GWAS to identify positional candidates, we acknowledge that prioritizing genes based on functional plausibility is crucial for downstream validation. To address this, we evaluated all overlapping candidate genes (Table 4, Table 5, Table 6 and Table 7) by integrating their functional annotations, homology information, and relevance to pericarp biology.

Based on this evaluation, we categorized the candidates into three tiers:

High-Priority Candidates with Direct Functional Links: This category includes genes whose known molecular functions can be directly linked to the cell wall biosynthesis, mechanical strength, or metabolic pathways underlying our measured traits. The most prominent example is *Zm00001eb314860*, annotated as a *bZIP* transcription factor. Its significant annotation in GO terms related to the positive regulation of biosynthetic processes strongly suggests a regulatory role in pathways determining pericarp structure and composition, making it our top candidate for future functional studies.

Candidates with Inferred or Indirect Functional Potential: This group comprises genes with unclear annotations but for which plausible functions can be inferred. For instance, *Zm00001eb084710* (isopentenyl transferase 2) near a thickness-associated SNP may be involved in cytokinin or gibberellin metabolism, potentially influencing cell division and expansion in the pericarp. Similarly, *Zm00001eb399220* (glutamate decarboxylase 4) could modulate gama-aminobutyric acid levels, which has been linked to stress responses and cell wall modification in plants.

Positional Candidates Requiring Further Evidence: Several genes listed currently have no informative annotation or clear homologs with known roles in relevant pathways. Their candidacy relies solely on physical proximity to significant SNPs. While they remain part of the associated intervals, their biological significance awaits validation through complementary approaches.

## 3. Discussion

### 3.1. Extensive Phenotypic Variation and Novel Genetic Loci Revealed by GWAS

Phenotypic characterization and genome-wide association study (GWAS) were conducted on pericarp traits using a panel of 196 fresh-eating maize inbred lines. This panel exhibits a rich genetic background, including various types such as sweet corn and waxy maize. Extensive variation was observed for pericarp thickness (ranging from 0.30 to 1.16 mm) and rupture strength (ranging from 213.13 to 2087.07 g), with relatively high coefficients of variation (27.42% for pericarp thickness and 28.06% for rupture strength). This provided the necessary genetic variation foundation for association analysis. Correlation analysis revealed a significantly positive correlation between pericarp thickness and rupture strength, while rupture strength showed a significantly negative correlation with brittleness. This suggests that materials selected for different market demands may possess distinct pericarp structures. The range of pericarp thickness variation observed in this study is comparable to that reported for Korean maize landraces [5], indicating that our population is representative in terms of key phenotypic diversity and sufficient to capture the allelic variation controlling this trait.

By integrating phenotypic data from 2023 and 2024 using Best Linear Unbiased Prediction (BLUP) and employing the FarmCPU model for GWAS, we successfully identified multiple significant SNP loci and candidate genes associated with pericarp thickness, rupture strength, and brittleness. Notably, most of the significant loci identified in this study did not overlap with known major QTL regions for pericarp thickness located on chromosome 2. For example, previous research indicated that overexpression of genes within the bin2.03 genomic region may positively regulate pericarp thickness [17]. Additionally, two candidate genes, *Zm00001d001964* and *Zm00001d002283*, have been screened as potential regulators of this trait [18]. Our results revealed novel genetic loci, suggesting that this investigation may have uncovered genetic factors specific to pericarp traits associated with the eating quality of this germplasm population.

### 3.2. Functional Analysis of ZmbZIP130 as a Key Candidate Gene

Among the numerous candidate genes, *Zm00001eb314860* (annotated as *ZmbZIP130*) is a particularly noteworthy pleiotropic candidate. GO functional annotation revealed that this gene was significantly enriched in a series of terms related to the “positive regulation of biosynthetic process” (e.g., GO:0045893, GO:0010558). Specifically, the term “positive regulation of macromolecule biosynthetic process” (GO:0010558) implies that this gene may activate the expression of genes involved in synthesizing cell wall components, such as cellulose and lignin. This provides a molecular clue for explaining its potential influence on pericarp thickness and rupture strength. Concurrently, the broader term “positive regulation of biosynthetic process” (GO:0009891) also suggests its potential involvement in regulating the synthesis of secondary metabolites like sugars, which may indirectly affect quality traits such as pericarp brittleness or flavor. This analysis connects the genetic locus identified by GWAS to a potential transcriptional regulatory function and constructs a plausible hypothesis for how this function influences specific pericarp traits, thereby providing clear direction for subsequent functional validation studies.

*ZmbZIP130* belongs to the *bZIP* transcription factor family. Plant *bZIP* transcription factors are characterized by conserved structural features and form hierarchical regulatory networks by interacting with signaling pathways such as light, ABA, and ethylene, thereby precisely coordinating key quality traits including fruit appearance, texture, nutrition, and seed storage compounds [19]. Systematic functional analyses in other crops support the pivotal role of the *bZIP* family. Recent studies highlight their core functions in controlling key fruit quality traits such as coloration, texture, and flavor, with molecular mechanisms elucidated across multiple crops [20,21].

For instance, the light-induced factor *PybZIPa*, identified in pear (*Pyrus pyrifolia*), exhibits a unique multi-pathway regulatory mechanism distinct from model species, precisely controlling pericarp pigmentation [22]. In studies on mango postharvest “soft-nose disorder,” *bZIP* was identified as one of the core transcription factor families regulating texture aberrations. It forms a complex regulatory network with the ERF and NAC families, synergizing with abscisic acid (ABA) and ethylene signaling pathways to co-regulate starch degradation and sucrose accumulation, ultimately driving pulp texture softening [23]. In banana fruit ripening, phosphorylation modification of *bZIP* regulates the expression of aroma compound biosynthesis-related genes (e.g., *MaLOX1*), influencing fruit flavor [24,25]. Research on kiwifruit (*Actinidia chinensis* Planch.) demonstrated that the cold stress-activated *bZIP* factor AcePosF21 interacts with the *R2R3-MYB* transcription factor *AceMYB102*, directly binding to the promoter of the vitamin C synthesis gene *AceGGP3* to enhance ascorbic acid (AsA) accumulation, coupling cold resistance with nutritional quality enhancement [26]. Genomic analysis of castor bean (*Ricinus communis*) identified seed-specific *bZIP* members (e.g., ABF subgroup) regulating oil synthesis via ABA signaling. This mechanism was validated in maize: the *bZIP* factor ZmbZIP29 responds to ABA, is phosphorylated by *SnRK2.2* kinase, and regulates the core factor *Opaque2* (*O2*), influencing grain protein content and hundred-kernel weight [27]. These cross-species evidences collectively confirm the evolutionarily conserved function of *bZIP* in processes like seed storage accumulation. Based on the above evidence, we speculate that *ZmbZIP130* may play a central role in maize pericarp development and quality formation through similar regulatory networks.

### 3.3. Genotype-by-Environment Interaction, Analytical Strategy, and Study Limitations

The phenotypic data in this study were derived from two-year trials conducted at the same location but under markedly different climatic conditions. The pollination and grain-filling period in 2023 was characterized by excessive rainfall and low temperatures, whereas the same period in 2024 experienced drought and high temperatures. This inter-annual climatic variation is the primary reason for the differences in the number and partial positions of significant association loci detected between the two years. Environmental factors likely influence pericarp development by regulating physiological processes such as cell wall biosynthesis and hormonal balance, demonstrating significant genotype-by-environment interaction. Consequently, this study employed BLUP values, which integrate data from both years, for the association analysis to identify genetic loci with stable expression across varying environments. The differences in annual results precisely underscore the importance of multi-environment evaluation and the BLUP model in identifying robust genetic signals. Simultaneously, the multi-year data and BLUP analysis mitigated random errors, and the consistency between years supports the reproducibility of the measurements.

This study, however, has limitations. The moderate population size (*n* = 196) may constrain the power to detect rare alleles or loci with minor effects. Furthermore, candidate genes were primarily selected based on physical proximity and functional annotation; whether they possess direct regulatory roles over pericarp traits requires further experimental validation through approaches such as gene editing, expression analysis, or transgenics. Future research should focus on elucidating the upstream and downstream regulatory pathways of *bZIP* across different crops to accelerate the development of high-quality, stress-resistant cultivars.

## 4. Materials and Methods

### 4.1. Plant Materials

A total of 196 experimental materials (Supplementary Table S2) were provided by the Maize Research Institute of the Jilin Academy of Agricultural Sciences. This collection included 21 sweet corn inbred lines and 175 waxy corn inbred lines, comprising 95 white waxy maize, 49 yellow waxy maize, 25 purple waxy maize, 6 black waxy maize, 12 white sweet maize, and 9 yellow sweet maize accessions.

### 4.2. Experimental Methods

#### 4.2.1. Phenotypic Identification of Pericarp-Related Traits

The experimental materials were sown over two years (2023 and 2024) at the breeding trial field of the Jilin Academy of Agricultural Sciences in Gongzhuling City, Changchun, Jilin Province. A randomized block design was adopted, with each material planted in 4 rows, each row being 5 m in length. Based on the days after pollination (23 days for sweet corn and 25 days for waxy corn), three well-filled self-pollinated ears from the central two rows were harvested for the evaluation of pericarp-related phenotypic traits [28].

Pericarp Thickness Measurement: Ten kernels were carefully peeled from the middle section of each ear using forceps. The pericarp from the top of the kernels was then precisely excised using forceps and a surgical blade, and sliced into thin sections. Pericarp thickness was measured using a digital outside micrometer (CHILON, Chengdu, China). The micrometer’s measuring surfaces were cleaned and calibrated to zero before each use. The thimble was rotated counterclockwise to open the measuring faces wider than the sample. The pericarp section was placed horizontally on the micrometer’s anvil (stationary face), and the thimble was rotated clockwise until a slipping sound was heard, indicating firm contact. The reading was then recorded. The average value from the measurements was calculated and used as the pericarp thickness for each plant [18].

Puncture Test for Tenderness: Kernel tenderness was assessed using a Stable Micro Systems TA.XT Plus texture analyzer (Stable Micro Systems, London, England) equipped with a 2 mm P/2E probe. Puncture tests were performed on individual kernels at 25 °C to determine pericarp rupture strength and brittleness. Each measurement was replicated three times, and the average value was used for subsequent analysis [29,30]. Kernels were selected from the middle section of the ear to minimize variation caused by different attachment positions on the cob. Each maize inbred line was measured with three replicates. The ear was securely fixed on the texture analyzer’s loading platform, and the measurement point was consistently positioned at the central region of the kernel’s crown.

The selection of pericarp rupture strength and brittleness as key traits was based on their established relevance to sensory quality in fresh-eating maize. Rupture strength reflects the mechanical resistance of the pericarp, directly influencing mouthfeel and postharvest durability, while brittleness quantifies the ease of fragmentation during mastication. These metrics are derived from standardized puncture tests using a texture analyzer (Stable Micro Systems TA.XT Plus with a P/2E probe), consistent with methods applied in fruit quality studies for crops such as melon [31]. To minimize measurement errors, the texture analyzer was calibrated before each batch of tests, and all kernels were sampled from the central section of the ear to reduce positional variation. Each measurement was repeated three times, and the average value was used for analysis. The coefficient of variation (CV) for each trait was within the acceptable range reported for similar fruit texture studies.

Data processing and statistical analyses were conducted using Microsoft Excel 2018 and IBM SPSS Statistics 23 software [32]. In our study, descriptive statistical analysis was performed on the phenotypic values of three pericarp traits (pericarp thickness, pericarp rupture strength, and pericarp brittleness), calculating the mean, standard deviation, range of variation, and coefficient of variation. Subsequently, Pearson correlation coefficient analysis was conducted to assess the pairwise correlations among the three traits.

#### 4.2.2. Genotyping of the Natural Population

Genotyping analysis was performed using Boruidi’s core genotyping technology—Genotyping by Target Sequencing (GBTS) [33]. A total of 196 fresh-eating maize inbred lines were used as experimental materials. The gene chip GenoBaits Maize 45K Panel was constructed for genotyping these accessions [34]. Raw sequencing reads were filtered using fastp software (v0.23.4) with the following steps [35,36]:

1. Removal of paired-end reads containing > 10% N bases relative to read length.
2. Removal of paired-end reads with >40% low-quality bases (Q ≤ 20).

Clean reads obtained after quality control were aligned to the reference genome sequence B73 RefGen_v5 using BWA software (v0.7.17) [37,38]. Variant calling and SNP extraction were performed using GATK (v3.5-0-g36282e4) [39], with SNPs exhibiting coverage depth < 5× filtered out [40]. Ultimately, genome-wide SNPs were obtained for subsequent analysis.

#### 4.2.3. Genome-Wide Association Study (GWAS)

In this study, Best Linear Unbiased Prediction (BLUP), a statistical method based on linear mixed models for predicting random effects, was employed in association analysis. GWAS was performed separately using phenotypic data from 2023, 2024, and BLUP values [41]. The formula of the linear model is:

$$Y_{ijk}=\mu +L_{i}+S_{j}+Y_{k}+{\left(LS\right)}_{ij}+{\left(LY\right)}_{ik}+\varepsilon_{ijk}$$

$Y_{ijk}$: Observation for genotype *i*, location *j*, and year *k.*

μ: Overall mean.

$L_{i}$: Random effect of genotype (Line). $N(0,\sigma_{L}^{2})$.

$S_{j}$: Random effect of location. $N(0,\sigma_{S}^{2})$.

$Y_{k}$: Random effect of year. $N(0,\sigma_{Y\;}^{2})$.

${\left(LS\right)}_{ij}$: Random interaction effect of genotype × location. $N(0,\sigma_{LS}^{2})$.

${\left(LY\right)}_{ik}$: Random interaction effect of genotype × year. $N(0,\sigma_{LY}^{2})$.

$\varepsilon_{ijk}$: Residual term (random error). $N(0,\sigma_{\varepsilon }^{2})$.

The broad-sense heritability calculation formula is:

$$h^{2}=\frac{\sigma_{L}^{2}}{\sigma_{L}^{2}+\frac{\sigma_{LS}^{2}}{2}+\frac{\sigma_{LY}^{2}}{2}+\frac{\sigma_{\varepsilon }^{2}}{4}}$$

$\sigma_{L}^{2}$: Genetic variance of genotype;

$\sigma_{LS}^{2}$: Variance of genotype × location interaction;

$\sigma_{LY}^{2}$: Variance of genotype × year interaction;

$\sigma_{\varepsilon }^{2}$: Residual variance.

Genotyping data were filtered and formatted using PLINK software [42], followed by genotype imputation using TASSEL 5.0 and calculation of Neighbor-Joining (NJ) genetic distances [43]. Population structure analysis was conducted with ADMIXTURE 1.3.0 using identified SNP markers, with K-values ranging from 1 to 10; each K-value underwent 10 independent iterations, and the optimal K-value was determined based on minimal cross-validation error [44,45]. Principal Component Analysis (PCA) and kinship analysis were performed using TASSEL 5.0 and R software, with results visualized to delineate subpopulation characteristics of inbred lines [46]. Linkage Disequilibrium (LD) decay distance was determined using TASSEL 5.0, defined as the chromosomal distance at which the average paired correlation coefficient (r^2^) dropped to half its peak value [47]. Genome-wide association analysis was implemented via the FarmCPU model incorporating kinship and PCA covariates, identifying significant trait-associated SNPs using a threshold of −log_10_(*p*) > 3.5 and MAF (Minor Allele Frequency) > 0.05; candidate intervals for significant loci were subsequently defined based on association results [48,49]. In plant GWAS studies, particularly those involving natural populations or germplasm collections of comparable scale, a significance threshold of −log_10_(*p*) > 3.5 has been widely adopted as a conventional and practical cutoff [50,51,52]. This threshold is chosen to balance the statistical power for detecting loci with moderate to weak effects against the need to control false positives in materials characterized by complex population structure. Candidate genes were screened within 50 kb upstream and downstream regions of significant SNPs identified by GWAS. To control for the potential confounding effects of population structure on the association analysis, the FarmCPU model employed in this study incorporated a kinship matrix (K matrix) calculated from genome-wide SNPs and the first three principal components (PCs) as covariates. This approach effectively corrects for population stratification and reduces false-positive associations.

#### 4.2.4. GO Functional Annotation of Pericarp-Related Traits

During this analysis, Entrez IDs were used instead of original gene identifiers; partial original IDs were filtered out due to the lack of matching Entrez IDs during the conversion process, resulting in a refined set of pericarp-related candidate genes [53].

The analysis was performed using the clusterProfiler R package (v4.0.3), which relied on the maize annotation database (AH117408) from AnnotationHub (https://annotationhub.bioconductor.org/ahid/AH117408 (accessed on 10 November 2025)). Fisher’s exact test was employed to compare the distribution of GO terms between the target gene set and the background gene set. The entire maize genome Entrez ID set was used as the background. Multiple testing correction was applied using the default Benjamini–Hochberg (FDR) method, with significance thresholds set at a *p*-value of 0.05 and a Q-value of 0.01.

## 5. Conclusions

In this study, descriptive statistics and correlation analysis of phenotypic data from 196 fresh corn inbred lines revealed a significant positive correlation between pericarp thickness and pericarp rupture strength, while pericarp rupture strength showed a significant negative correlation with pericarp brittleness. No correlation was observed between pericarp thickness and pericarp brittleness. Genotyping was performed using the GenoBaits Maize 45K Panel SNP chip, and genome-wide association analysis (GWAS) was conducted for pericarp-related traits. Based on multi-year pericarp thickness data, 19 overlapping SNPs were identified, and 47 candidate genes associated with pericarp thickness were annotated. For multi-year pericarp rupture strength data, 2 overlapping SNPs were screened, yielding 7 candidate genes related to pericarp rupture strength. From multi-year pericarp brittleness data, 1 overlapping SNP was identified, along with 4 candidate genes associated with pericarp brittleness. Annotation analysis of overlapping loci for pericarp-related trait candidate genes indicated that the *bZIP* transcription factor family member *ZmbZIP130* may function as a pleiotropic gene regulating pericarp-related traits.

## Figures and Tables

**Table 1:** Descriptive statistics of quality traits of different fresh corn.

Trait	Mean	Standard Deviation	Variation Rang	Coefficient of Variation (%)	Heritability (%)
Pericarp Thickness (mm)	0.62	0.17	0.30–1.16	27.42	20.02
Pericarp Rupture Strength (g)	1045.54	293.43	213.13–2087.07	28.06	17.81
Pericarp Brittleness (g.sec)	2.77	0.40	1.60–4.50	14.44	26.57

**Table 2:** Pearson Correlation Analysis of Pericarp-Related Traits.

	Pericarp Thickness	Pericarp Rupture Strength	Pericarp Brittleness
Pericarp Thickness	1		
Pericarp Rupture Strength	0.140 *	1	
Pericarp Brittleness	0.125	−0.152 *	1

Note: * indicates significant correlation at the 0.05 level.

**Table 3:** Distribution information of SNP on each chromosome.

Chromosome	SNPs	Density (Per Mbp)
Chr1	40,542	134.47
Chr2	30,861	129.72
Chr3	33,280	143.32
Chr4	26,913	111.16
Chr5	28,314	129.88
Chr6	21,007	124.01
Chr7	22,127	125.15
Chr8	21,622	123.27
Chr9	20,633	131.42
Chr10	19,254	128.7
Average	26,455.3	128.43

**Table 4:** Overlapping Significant SNPs and Candidate Genes for Pericarp Thickness Identified in GWAS Analyses of 2023, 2024, and BLUP.

Chromosome	Position	Reference	Alternate	Candidate Genes	Annotations
1	202,205,975	G	A	*Zm00001eb037610*	NC domain-containing protein-related
				*Zm00001eb037620*	Uncharacterized
				*Zm00001eb037630*	photosystemI2
				*Zm00001eb037640*	Activator of 90 kDa heat shock protein ATPase
	202,206,517	G	A	*Zm00001eb037630*	photosystemI2
				*Zm00001eb037640*	Activator of 90 kDa heat shock protein ATPase
2	64,578,153	G	A	*Zm00001eb084700*	Uncharacterized
				*Zm00001eb084710*	isopentenyl transferase2
	198,486,886	C	T	*Zm00001eb102550*	pyruvate dehydrogenase3
3	160,120,677	C	T	*Zm00001eb142850*	L-type lectin-domain containing receptor kinase IX.1
				*Zm00001eb142860*	Uncharacterized
	160,157,364	A	G	*Zm00001eb142850*	L-type lectin-domain containing receptor kinase IX.1
				*Zm00001eb142860*	Uncharacterized
	160,346,446	C	A	*Zm00001eb142890*	Uncharacterized
				*Zm00001eb142900*	Uncharacterized
				*Zm00001eb142910*	Uncharacterized
				*Zm00001eb142920*	Uncharacterized
	160,368,430	T	C	*Zm00001eb142890*	Uncharacterized
				*Zm00001eb142900*	Uncharacterized
				*Zm00001eb142910*	Uncharacterized
				*Zm00001eb142920*	Uncharacterized
	161,998,093	A	T	*Zm00001eb143120*	nucleobase: cation symporter10
				*Zm00001eb143130*	nucleobase: cation symporter10
				*Zm00001eb143150*	uncharacterized
				*Zm00001eb143160*	uncharacterized
6	160,083,262	T	C	*Zm00001eb288790*	uncharacterized
				*Zm00001eb288800*	auxin import carrier4
	164,570,952	G	T	*Zm00001eb290360*	protein transporter
				*Zm00001eb290370*	uncharacterized
				*Zm00001eb290380*	uncharacterized
				*Zm00001eb290390*	uncharacterized
7	45,582,095	G	A	*Zm00001eb306680*	protein binding protein
8	64,175,962	A	G	*Zm00001eb342510*	mov34/MPN/PAD-1 family protein pseudogene
9	5,510,195	G	C	*Zm00001eb372240*	uncharacterized
	5,991,255	C	A	*Zm00001eb372390*	uncharacterized
				*Zm00001eb372400*	carboxyesterase2
	150,496,166	A	T	*Zm00001eb399220*	glutamate decarboxylase4
				*Zm00001eb399230*	Pentatricopeptide repeat-containing protein
				*Zm00001eb399240*	leunig-related14
10	5,365,786	G	A	*Zm00001eb406820*	dihydrodipicolinate decarboxylase1
				*Zm00001eb406830*	DNA-3-methyladenine glycosylase4
				*Zm00001eb406840*	uncharacterized
				*Zm00001eb406850*	uncharacterized
				*Zm00001eb406860*	uncharacterized
				*Zm00001eb406870*	uncharacterized
	87,284,261	T	G	*Zm00001eb417200*	glycogen synthase kinase7
	120,473,230	A	G	*Zm00001eb417210*	uncharacterized

**Table 5:** Overlapping Significant SNPs and Candidate Genes for Pericarp Rupture Strength Identified in GWAS Analyses of 2023, 2024, and BLUP Values.

Chromosome	Position	Reference	Alternate	Candidate Genes	Annotations
5	163,983,546	C	G	*Zm00001eb241080*	Os02g0478550-like protein
				*Zm00001eb241090*	ribosomal protein S27b
				*Zm00001eb241100*	DUF679 domain membrane protein 7
				*Zm00001eb241110*	RS21-C6 protein
10	133,474,083	C	T	*Zm00001eb426710*	uncharacterized
				*Zm00001eb426720*	pentatricopeptide repeat protein 513
				*Zm00001eb426730*	integral membrane protein like protein

**Table 6:** Overlapping Significant SNPs and Candidate Genes for Pericarp Brittleness Identified in GWAS Analyses of 2023, 2024, and BLUP Values.

Chromosome	Position	Reference	Alternate	Candidate Genes	Annotations
9	6,307,872	G	A	*Zm00001eb372460*	uncharacterized
				*Zm00001eb372470*	intramolecular lyase activity
				*Zm00001eb372480*	osmotin-like protein
				*Zm00001eb372490*	TCP-transcription factor 9

**Table 7:** Candidate genes screened and annotations.

Candidate Genes	Overlapping	Name	Annotations
*Zm00001eb314860*	PRS BLUP + PT BLUP	*ZmbZIP130*	*bZIP130* transcription factor
*Zm00001eb314870*	PRS BLUP + PT BLUP		non-coding RNA
*Zm00001eb314880*	PRS BLUP + PT BLUP		uncharacterized
*Zm00001eb415550*	2023 PB + PT BLUP	*ZmCCR4*	serine/threonine protein kinase *CCR4*
*Zm00001eb415560*	2023 PB + PT BLUP	*ZmCCR4*	serine/threonine protein kinase *CCR4*

Note: PT: Pericarp thickness. PRS: Pericarp rupture strength. PB: Pericarp brittleness.

## Data Availability

The original contributions presented in this study are included in the article. Further inquiries can be directed at the corresponding author.

## Supplementary Materials

The following supporting information can be downloaded at: https://www.mdpi.com/article/10.3390/plants15010074/s1, Table S1: Data on fruit peel-related traits; Table S2: Types and Quantities of Experimental Materials.
