# Supplementary material for: Genome-Wide Association Study Dissects the Genetic Architecture of Pericarp Traits in Fresh-Eating Maize
Source: Plants (Basel). 2025 Dec 25;15(1):74. doi: 10.3390/plants15010074 (PMC12787704; doi:10.3390/plants15010074)
Supplement: Supplementary file 1 [file plants-15-00074-s001.zip › Supplementary File Table S2.pdf]

Table S2. Types and Quantities of Experimental Materials

<table>
<tr><th>White Waxy Maize</th><th>Yellow Waxy Maize</th><th>Purple Waxy Maize</th><th>Black Waxy Maize</th></tr>
<tr><td>95 samples</td><td>49 samples</td><td>25 samples</td><td>6 samples</td></tr>
<tr><td rowspan="4">A8、A9、A10、A11、A12、A13、A14、A15、A16、A17、A18、A19、A20、A26、A35、A38、A39、A41、A44、A45、A51、A52、A55、A56、A58、A59、A60、A61、A63、A64、A70、A71、A72、A73、A76、A77、A78、A79、A82、A83、A84、A85、A86、A87、A88、A97、A98、A99、A100、A101、A102、A103、A104、A105、A106、A107、A116、A117、A119、A120、A121、A122、A123、A124、A125、A126、A127、A129、A130、A131、A136、A137、A150、A151、A152、A153、A154、A155、A156、A158、A160、A166、A167、A168、A169、A172、A173、A175、A176、A181、A187、A189、A190、A191、A192</td><td rowspan="4">A6、A7、A21、A25、A27、A28、A29、A30、A37、A42、A46、A48、A49、A53、A62、A89、A90、A91、A92、A93、A94、A95、A96、A108、A109、A110、A111、A112、A113、A115、A118、A128、A132、A133、A134、A135、A138、A139、A140、A141、A142、A143、A159、A161、A164、A165、A178、A179、A185</td><td>A22、A23、A24、A36、A40、A43、A47、A50、A54、A65、A66、A67、A68、A75、A80、A81、A144、A145、A147、A148、A171、A174、A177、A180、A188</td><td>A57、A69、A74、A146、A170、A186</td></tr>
<tr><th>White Sweet Maize</th><th>Yellow Sweet Maize</th></tr>
<tr><td>12 samples</td><td>9 samples</td></tr>
<tr><td>A1、A2、A3、A5、A31、A33、A34、A149、A162、A194、A195、A196</td><td>A4、A32、A114、A157、A163、A182、A183、A184、A193</td></tr>
</table>
